# Body camera footage leads to lower judgments of intent than dash camera footage

**DOI:** 10.1073/pnas.1805928116

**Published:** 2019-01-07

**Authors:** Broderick L. Turner, Eugene M. Caruso, Mike A. Dilich, Neal J. Roese

**Affiliations:** ^a^Kellogg School of Management, Northwestern University, Evanston, IL 60208;; ^b^Anderson School of Management, University of California, Los Angeles, CA 90095;; ^c^Foresight Reconstruction, Inc., Morton Grove, IL 60053

**Keywords:** body camera, dash camera, attribution, intention, visual salience

## Abstract

Police departments use body-worn cameras (body cams) and dashboard cameras (dash cams) to monitor the activity of police officers in the field. Video from these cameras informs review of police conduct in disputed circumstances, often with the goal of determining an officer’s intent. Eight experiments (*N* = 2,119) reveal that body cam video of an incident results in lower observer judgments of intentionality than dash cam video of the same incident, an effect documented with both scripted videos and real police videos. This effect was due, in part, to variation in the visual salience of the focal actor: the body cam wearer is typically less visually salient when depicted in body versus dash cam video, which corresponds with lower observer intentionality judgments. In showing how visual salience of the focal actor may introduce unique effects on observer judgment, this research establishes an empirical platform that may inform public policy regarding surveillance of police conduct.

## Significance

Surveillance video from body cams and dash cams is increasingly used by police organizations to enhance accountability, and yet little is known about their effects on observer judgment. Across eight experiments, body cam footage produced lower judgments of intent in observers than did dash cam footage, in part, because the body cam (vs. dash cam) visual perspective reduces the visual salience of the focal actor. This research informs public policy regarding the interpretation of video surveillance methods of police conduct.

In 2014, a grand jury decided not to indict police officer Darren Wilson for the fatal shooting of Michael Brown, an unarmed teenager. Surveillance video of the shooting was not available. Due, in part, to such controversial police action, communities are demanding greater accountability of police officers, with both body-worn cameras and dashboard cameras seen as a key means of doing so (1). Many major police forces in the United States and across the globe now mandate or plan to mandate body cam use, and a majority of police forces now equip their vehicles with dash cams (2). Grand juries can consider both types of footage in deliberations to indict (3). Underlying the legal consideration of an officer engaged in a controversial act is (among other factors) the attributional judgment of intentionality, i.e., the extent to which an individual acts with the goal to produce a specific outcome (4). Despite the widespread use of surveillance video, little is known about its specific impact on intentionality judgments.

Whereas legal scholarship contends that surveillance video, like body cam video, may provide an accurate depiction of events that can protect officers in a court of law against unwarranted accusations (5), the effects of this video on observers’ judgments are more varied. For instance, a study using mock jurors found that their preexisting attitudes toward the police influenced their interpretations of an officer’s actions whether or not they saw the body cam footage of the event (6). However, other work found that when participants see body cam footage in conjunction with an officer’s report that contradicts a suspect’s report, participants viewed the officer more positively, the suspect more negatively, and were more likely to justify the use of force (7). We attempt to clarify and expand on existing literature in this area by conducting a systematic investigation of the impact of these two types of surveillance video on observer judgments of intent.

The present research used an experimental approach to examine variation in observer judgment as a function of witnessing the same episode via body cam or dash cam footage (i.e., same duration, start time, and end time). Conceptually, we defined body cam as a first-person visual perspective that captures an incident from the viewpoint of a focal actor with few visual cues of the actor’s body. We defined dash cam as the visual perspective that captures the same incident from a third-person perspective at a similar height as, but broader depth than, body cam, thus rendering the actor’s body more visually prominent.

We propose that the visual salience of actors in videos influences subsequent intentionality judgments of those actors by observers, and because body cam footage typically features diminished visual salience of a focal actor, observers’ judgments of the intentionality of that actor’s actions will also be diminished. In general, attention is naturally drawn to the human form (8, 9). Observers tend to attribute intentionality as a function of the visual salience of, and hence attention to, the focal actor. When an actor is visually deemphasized (e.g., by way of manipulations of observer seating position or video camera angle), judgments of the intentionality of that actor are reduced (10–12). This effect occurs mainly at encoding as opposed to retrieval and has implications for legal judgment, for example, in the use of videotaped police interrogations later used in courts of law (13). We tested this visual salience account by manipulating the presence of visual indications of the body cam wearer (e.g., arms). If the visual salience account is sound, then body cam footage in which body parts are visible for longer durations should result in intentionality judgments that are more similar to those resulting from dash cam footage. Importantly, this account does not presume that all body cam videos lack visual cues of the focal actor. Rather, we conceptualize the presence and duration of such visual cues as a continuum, with body cam videos featuring on average fewer and less frequent visual cues than dash cam videos.

## Police-Involved Video Analysis.

**Fig. 1.**
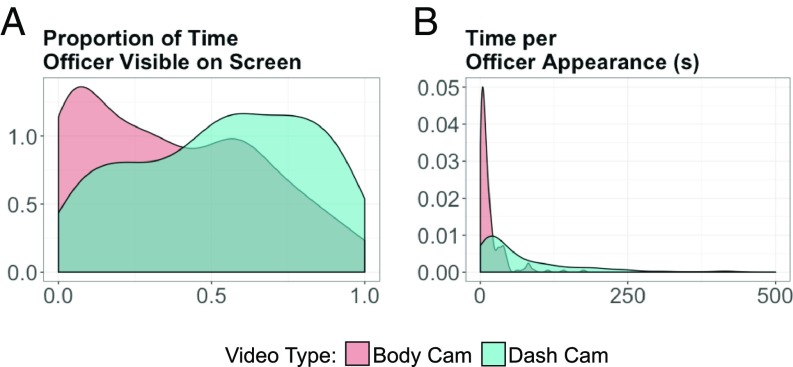
Visual salience indicators; proportion of time officer visible on screen (*A*) and the time per officer appearance (*B*) as a function of body cam vs. dash cam. Note that the filled curves are estimates of the probability density functions of each of the variables by video type, fit using kernel density estimation.

Confirmation of this conceptualization came from a systematic analysis of publicly available police videos. We used 279 online respondents to assess videos [mean = 2.49 videos coded (SD = 4.34)], specifying the points in time that a focal police officer (i.e., the body cam wearer) was visible onscreen (i.e., moved on and off screen). Our video sample comprised 393 publicly available police videos (206 body cam vs. 187 dash cam) identified by a search of the YouTube Application Programming Interface (API) for videos of police-involved incidents. The body cam wearer in body cam (vs. dash cam) videos was visible for proportionally less time onscreen (means = 0.37 vs. 0.54; SDs = 0.29, 0.28), *t*(391) = 5.90, *P* < 0.001; and more frequently moved into and out of the field of view (means = 9.46 vs. 2.19; SDs = 13.47, 2.03), *t*(391) = 7.30, *P* < 0.001; with each such appearance being comparatively briefer (means = 16.4 s vs. 77.0 s; SDs = 23.5, 98.0), *t*(391) = 8.63, *P* < 0.001 (Fig. 1). Thus, body cam footage contains fewer visual indicators of the focal wearer than does corresponding dash cam footage.

We also tested a second account as to why body cam (vs. dash cam) footage might result in lower intentionality judgments. According to a motivational account, observers of body cam footage may be more likely to engage in a process of perspective taking than observers of dash cam footage and, thus, adopt the motivational stance of the actor in question (14). As a result, the observer is motivated to avoid blame for negative outcomes and to accept praise for positive outcomes (15). Thus, because body cams, more so than dash cams, induce observers to take the actor’s perspective, and because most police videos used in court depict negative outcomes, the resulting motivation is to avoid blame for the negative outcome, which reduces intentionality judgments relative to dash cam videos.

Stimuli included both staged and real police videos. To enhance experimental control, all videos were presented without sound. Unless noted, each video was played on a continuous loop. All participants watched the entirety of a video at least once. Although our software implementation was not designed to measure number of viewings, there was no difference across conditions in total viewing time (*P* = 0.95). The key dependent measure was participants’ intentionality judgments rendered using standard rating scales.

## Experiment 1.

Experiment 1 tested whether body cam (vs. dash cam) influences intentionality judgments. Each participant viewed three video scenes filmed either via body cam or dash cam (as a between-participant block). These videos depicted real police-involved incidents. Two videos depicted shootings (both 10 s in length); the third depicted an officer breaking a suspect’s car window (43 s). Dependent measures included intentionality judgment of the police officers, as well as blame and recommended punishment.

We found that body cam participants gave lower intentionality ratings than did dash cam participants, *t*(248) = 3.39, *P* < 0.001, *d* = 0.43; as well as lower ratings of blame, *t*(248) = 4.98, *P* < 0.001, *d* = 0.63; and recommended punishment, *t*(248) = 5.97, *P* < 0.001, *d* = 0.76 (see Table 1 for means).

**Table 1.** Means (SDs) of observer intentionality ratings in all experiments

Experiment	*N*	Condition	Mean (SD)
1	250	Body cam	5.10 (1.24)
		Dash cam	5.61 (1.13)
2	105	Body cam	2.66 (1.32)
		Dash cam	3.25 (1.54)
3	220	Body cam	2.79 (1.31)
		Dash cam	3.58 (1.85)
4	348	Body cam-perspective taking	5.12 (1.59)
		Dash cam-perspective taking	5.94 (1.30)
		Body cam-control	5.26 (1.49)
		Dash cam-control	5.82 (1.48)
5	260	Body cam-neutral outcome	5.50 (1.10)
		Dash cam-neutral outcome	5.73 (1.28)
		Body cam-negative outcome	5.39 (1.14)
		Dash cam-negative outcome	5.86 (1.04)
6	308	Body cam-obscured	4.97 (1.77)
		Dash cam	6.40 (1.16)
		Body cam-visible	6.46 (1.07)
7	425	Body cam-obscured	5.42 (1.60)
		Body cam-face	5.48 (1.62)
		Dash cam	6.61 (0.83)
		Body cam-visible	6.58 (0.84)
8	203	Report-only	2.10 (1.04)
		Body cam-report	3.09 (1.24)
		Dash cam-report	3.63 (1.60)

## Experiments 2 and 3.

Experiments 2 and 3 used staged videos (each 6 s) in which one actor bumped into another, recorded simultaneously by body cam and dash cam (a between-participant manipulation). The dependent measure was an intentionality rating (e.g., “Person B intentionally bumped into Person A”; 1 = strongly disagree, 7 = strongly agree). To control for variation in actor characteristics, we created two versions of each video scene, differing only in who wore the body cam and initiated the bump, randomized across participants. Body cams were positioned at chest height, whereas the dash cam was positioned at waist height and 3 m from the focal action.

To aid participants in recognizing which actor was which, an orienting video (3 s) established scene and viewpoint. Participants then viewed the target video and rated intentionality. Experiment 2 used male actors and Experiment 3 used female actors (video presented once). Viewing body cams (vs. dash cam) reduced intentionality ratings in Experiment 2, *F*(1, 101) = 4.39, *P* = 0.04, *d* = 0.41, and in Experiment 3, *F*(1, 216) = 12.94, *P* < 0.001, *d* = 0.29 (Table 1).

## Experiment 4.

This experiment tested the motivational account. We manipulated perspective taking using a standard procedure (14) alongside the manipulation of body cam versus dash cam (using two of three videos from Experiment 1). If body cam footage invites viewers to take the wearer’s perspective, then the perspective taking intervention should reduce differences in intentionality between the conditions. A manipulation check confirmed the success of the perspective taking manipulation. Intentionality ratings were lower in the body cam versus dash cam conditions, *F*(1, 341) = 17.09, *P* < 0.001, *d* = 0.47; perspective taking did not moderate this effect, *F*(1, 341) = 0.57, *P* = 0.45; nor was there a significant main effect of perspective taking, *F*(1, 341) = 0.001, *P* = 0.98 (Table 1). Thus, Experiment 4 did not support the motivational account.

## Experiment 5.

This experiment further tested the motivational account. When police videos depict negative outcomes, the motivation of the wearer may be to avoid blame; hence, judgments of intent may be lower in body cam videos relative to dash cam videos. Accordingly, we manipulated the valence of the incident alongside the manipulation of body cam versus dash cam (same stimuli as Experiment 4). The motivational account would predict that body cam (vs. dash cam) would involve lessened intentionality judgments for negative, but not neutral, outcomes.

We manipulated body cam versus dash cam on a between-participant basis and, orthogonally, whether incident valence was negative (an innocent person was injured) or neutral (a suspect was stopped). A manipulation check confirmed the success of the incident valence manipulation. Body cam resulted in lower intentionality ratings than dash cam, *F*(1, 256) = 5.09, *P* = 0.03; *d* = 0.33; incident valence did not moderate this effect, *F*(1, 256) = 1.02, *P* = 0.31; nor was there a significant main effect of incident valence, *F*(1, 256) = 0.04, *P* = 0.84 (Table 1). Thus, neither Experiment 4 nor 5 supported the motivational account.

## Experiment 6.

We propose that body cam footage lowers judgments of intentionality relative to dash cam footage because body cams typically contain fewer visual indicators of the focal actor, which decreases attention to that actor. We tested this account by manipulating the presence of visual indicators of the body cam wearer’s body (arms and feet). If the visual salience account is correct, the increased visual salience of the body cam wearer should mitigate any reduction in intentionality judgment by body cam (vs. dash cam).

For Experiment 6, we created new videos that manipulated body cam versus dash cam, but in addition, the body cam version contained either no visual cues of the wearer’s body (body cam-obscured condition) or did contain such visual cues, specifically, the body cam wearer’s arms or feet (body cam-visible condition). The videos depicted mundane incidents in which the actor was overtly intentional [tipping over a cup (2 s), dropping a magazine (3 s), kicking over a trashcan (3 s), and pulling down a stuffed animal (3 s)].

A mixed linear regression with the participant and the incident as random factors, and with viewpoint, incident, and their interaction as fixed factors, revealed a significant within-participant main effect of visual perspective on intentionality judgment (β = 1.47; SE = 0.11), *t* = 13.69, *P* < 0.001. The body cam-obscured condition yielded lower intentionality ratings than the dash cam condition, *t*(771) = 13.55, *P* < 0.001, *d* = 1.01, but also lower than the body cam-visible condition, *t*(820) = 13.62, *P* < 0.001, *d* = 0.97 (Table 1). Intentionality judgments did not differ between the body cam-visible and dash cam conditions, *t*(781) = −0.77, *P* = 0.44, *d* = 0.06. There was no main effect of incident (β = −0.16; SE = 0.17), *t* = −0.57, *P* = 0.63, nor was there an interaction between incident and visual perspective (β = 0.17; SE = 0.17), *t* = 0.97, *P* = 0.33.

## Experiment 7.

Experiment 7 added a condition to further test the visual salience account. If the mere presence of body parts onscreen mitigates the reduction in intentionality judgment by body cam (vs. dash cam), then other reminders of the presence of the body cam wearer might similarly mitigate the effect. Experiment 7 added a modified version of the body cam-obscured condition by including identifying information (picture and name) of the body cam wearer (body cam-face condition). If this visual reminder restores attention to the body cam wearer, then this condition might also mitigate the difference between body cam and dash cam video in intentionality judgments. In both experiments, participants viewed all four incidents, each independently randomized to show one viewpoint.

In Experiment 7, the same analysis with the additional body cam-face condition yielded a significant within-participant main effect of visual perspective on intentionality judgment (β = 0.53; SE = 0.08), *t* = 6.37, *P* < 0.001. Participants made lower intentionality judgments in the body cam-obscured versus the dash cam condition, *t*(882) = 13.86, *P* < 0.001, *d* = 0.93, replicating Experiment 6. Intentionality judgments did not differ between the body cam-visible condition and the dash cam condition, *t*(871) = 0.53, *P* = 0.60, *d* = 0.04. Contrary to our expectations, the body cam-face condition did indeed result in lower intentionality judgments than in the dash cam condition, *t*(821) = 12.83, *P* < 0.001, *d* = 0.88, and the body cam-face condition yielded higher intentionality ratings than the body cam-visible condition, *t*(814) = 12.37, *P* < 0.001, *d* = 0.85. Finally, there was no main effect of incident (β = 0.05; SE = 0.32), *t* = 0.14, *P* = 0.90, nor was there an interaction between incident and visual perspective (β = 0.24; SE = 0.21), *t* = 0.99, *P* = 0.26.

Experiments 6 and 7 support the visual salience account, in that the presence of visible body parts in bodycam footage mitigates the previously observed effect of body cam versus dash cam on intentionality judgments (Table 1). However, the attempt to provide additional evidence of this account with the body cam-face condition was unsuccessful. We speculate that static information about an actor’s identity (e.g., a face) matters less in this context than does dynamic imagery (e.g., the movement of the actor’s arms), because the latter conveys additional information about how the incident unfolds in real time, including subtle cues as to the actor’s mental state.

## Experiment 8.

To consider the applicability of the present research to police accountability, Experiment 8 tested intentionality judgments alongside binary legal decisions (indict vs. not indict). In the United States, the decision to indict a police officer is often made by a grand jury, a legal body comprising 16 to 23 citizens. If more than half decide to indict, the case goes to trial; otherwise, no charges result. We recruited participants who qualified for jury duty in Illinois (at least 18 y old; current state resident) from a field laboratory in Chicago, IL.

The experiment used a single incident to test variation across three between-participant conditions. All participants saw a redacted version of an actual police report describing a vehicle stopped in traffic. The police officer knocked on the car window, startling the driver, who then accelerated suddenly and crashed. In the report-only condition, participants read the report but saw no video. In the body cam-report and dash cam-report conditions, participants read the report and then viewed the corresponding video (70 s) of the incident. All participants then made intentionality judgments and four indictment decisions: assault, battery, aggravated battery, and official misconduct (Illinois Compiled Statutes: 720 ILCS 5/12-1, 5/12-3, 5/12-3.05, and 5/33-3, respectively).

Body cam-report participants gave lower intentionality ratings (mean = 3.09; SD = 1.24) than did dash cam-report participants (mean = 3.63, SD = 1.60), *t*(134) = 2.32, *P* = 0.02, *d* = 0.39, consistent with results from the previous experiments. However, the report-only condition resulted in lower intentionality ratings (mean = 2.10; SD = 1.04) than in both the body cam-report and dash cam-report conditions, *t*(135) = 5.00 and *t*(131) = 6.63; *P*s < 0.001; *d*s = 0.87 and 1.15. This latter finding was unexpected: we had expected to see lower (rather than greater) intentionality ratings in the body cam-report condition than in the report-only condition, which would have demonstrated that body cam footage reduces intentionality relative to a baseline in which no video is seen.

We next tested effects on the four indictment decisions. Official misconduct involves the lowest threshold of evidence to indict; it showed nonsignificant variation across conditions: body cam-report = 65.3%; dash cam-report = 79.4%; report-only = 78.5%; $\chi^{2}$ = 4.74; *P* = 0.09. By contrast, body cam-report participants were less likely to indict than were dash cam-report or report-only participants for each of assault: 48.6% vs. 70.6% vs. 73.41%, $\chi^{2}$ = 11.76, *P* = 0.003; battery: 52.8% vs. 69.1% vs. 75.9%, $\chi^{2}$ = 9.41, *P* = 0.009; and aggravated battery: 48.6% vs. 60.3% vs. 75.9%, $\chi^{2}$ = 12.10, *P* = 0.002.

To assess indictment decisions at the grand-jury level, we followed a previously established method (16) to create a bootstrap of 1,000 simulated grand juries of 16 jurors each (sampled from each condition). In Illinois, if fewer than nine jurors decide against indictment, the case does not go to trial. Simulated juries were less likely to send the case to trial in the body cam-report condition than in the dash cam-report or report-only condition, χ^2^(3, *n* = 2,666) = 145.80, *P* < 0.001. Inclusion of body cam footage resulted in diminished likelihood of indicting the police officer in three of the four charges, compared with both the dash cam-report and report-only condition. When body cam is included, the mean odds of indictment decrease compared with dash cam (odds ratio = 5.63).

Body cam (relative to dash cam) video may reduce observer punitiveness. However, several points of caution are warranted. First, although grand juries typically comply with indictment requests (17), police officers are rarely indicted for severe actions, such as police shootings (18). Between 2005 and 2011, 41 officers were charged with manslaughter or murder in connection with on-duty shootings; during the same period, about 2,700 justified homicides by police were reported to the Federal Bureau of Investigation (19). Second, participants made decisions individually (rather than in groups, as do real grand jurors). Group decision making may introduce further complexity that our paradigm cannot capture. Third, we observed a discrepant pattern between intentionality judgments and indictment decisions in two of our conditions: participants gave higher intentionality ratings in the body cam-report than report-only condition but were less likely to indict in the body cam-report than report-only condition. We had expected that higher intentionality judgments would lead to higher indictment decisions across all conditions, so this discrepancy is clearly at odds with our expectation.

To help clarify this discrepancy, we examined the relation between intentionality ratings and indictment decisions. When controlling for the four charges, intentionality only weakly predicted indictment decisions (β = 0.23; SE = 0.13; *z* = 1.85; *P* = 0.06). This result underscores how intent is only one of many inputs into indictment decisions. We speculate that the relatively weak relationship we observe in the current paradigm may be partly explained by differences in what people actually see in the video and what they imagined when reading the police report alone. Accordingly, we believe much more work is needed to understand this unpredicted pattern of results with respect to the report-only condition.

## Empirical Summary.

We conducted a metaanalysis across the present experiments (and confirm that we report here all experiments conducted to date) with the goal of specifying a mean effect size of body cam (and body cam-obscured) versus other camera conditions (dash cam, body cam-visible, body cam-face). Using a random-effects model that accounts for variation across experiments, the standardized estimate was significant (*r* = 0.30; 95% CI: 0.21–0.38; *z* = 6.30; *P* < 0.001).

As an omnibus test of the visual salience account, we used the procedure from the Police-Involved Video Analysis. The proportion of time on screen, number of appearances, and time per appearances were fixed factors predicting intentionality judgments for all 26 videos used in our experiments. The resulting model also contained a fixed factor for body cam (vs. other camera conditions). Individual video, nested within experiments, was a random factor and explained 19.1% of the variance. The overall model was significant (β_0_ = 4.16; SE = 0.81), *t* = 5.11, *P* < 0.001. Of the four fixed factors, only the proportion of time on screen factor predicted intentionality judgments (β = 1.45; SE = 0.34), *t* = 4.24, *P* = 0.001. Neither the number of appearances (β = −0.06; SE = 0.06), *t* = −1.10, *P* = 0.29, nor the time per appearance (β = −0.02; SE = 0.02), *t* = −1.15, *P* = 0.27, was significant. The fixed factor for body cam (vs. other camera conditions) was no longer significant (β = 0.15; SE = 0.30), *t* = 0.51, *P* = 0.62. In other words, we noted a robust effect of body cam versus dash cam on intentionality judgments across experiments, an effect due partly to variation in visual salience.

## Discussion

With calls to enhance the accountability of police through increased use of video, it is essential to understand the psychological processes underlying judgments by observers of those videos. Because video is an increasingly important source of evidence in criminal trials, the quality of legal decision making based on such video may be informed by empirical research. The present research compares the consequences of new video forms on the intentionality judgments made by observers. We find systematic evidence that body cam footage can result in lower observer intentionality judgments than does dash cam footage of the same incident. We further find that the visual salience of the body cam wearer accounts in part for this difference, such that greater visibility of the body cam wearer’s body corresponds with greater intentionality attributed to that wearer. In essence, the difference between body cam and dash cam reflects the impact of visual salience of actors on attributional judgment, an effect first documented by research in the 1970s (10–12).

If the difference between body cam and dash cam footage is interpreted as bias on the part of body cam, this research suggests that viewing body cam footage might make judgments by jurors and as well by the general public more lenient toward the body cam wearer (usually a police officer) than might otherwise be warranted. Experiment 8 indicates that body cam video might in some cases reduce the likelihood that grand juries will indict a police officer, compared with dash cam video or a written report of the incident. However, these findings contain an anomaly, such that the recommendation to indict was less likely in the body cam-report condition than in the report-only condition even though intentionality was rated higher in the former than the latter condition. Because many trials rely solely on verbal reports, it is critical for future work to determine how generalizable our findings are to the indictment decisions that juries routinely must make in the absence of video evidence.

Although body cam may introduce bias in observer judgment, dash cam may also introduce bias. That is, dash cam or any other video angle that emphasizes the visual salience of a focal actor may increase intentionality judgments regarding that focal actor. Such an effect would be compatible with the conception of correspondence bias, defined as the tendency of observers to overattribute an actor’s behavior to internal aspects, such as personality or intentions (20). One piece of evidence in the present research argues against this latter interpretation, namely that the manipulation to take the body cam wearer’s perspective did not alter intentionality judgments, as would be predicted from prior demonstrations that perspective taking reduces correspondence bias (21).

We acknowledge that different forms of surveillance video—whether via body cam, dash cam, or some other perspective—will vary on a host of important dimensions, even as they capture the same event. In the present research, we have identified one important dimension that is less common in body cam than dash cam footage, namely the visual salience of the focal actor. Although further research is needed to better specify this effect, the current work outlines a key process that underlies intentionality judgments, namely the visibility of the focal actor. Other future research directions include examination of whether the impact of visual perspective on observer judgment is qualified by the actor’s sex and race, or by presentational aspects such as camera shakiness, opportunity for repeat viewing, or presence versus absence of auditory cues. For society to benefit most from the greater transparency conferred by emerging forms of surveillance, these advances in technology require corresponding advances in our understanding of their effects on observer judgment.

## Materials and Methods

### Ethics Statement.

All experiments were approved by the Institutional Review Board at Northwestern University. All participants read and provided informed consent before completing the experiments.

### Open Science Statement.

Experiment materials and raw data are available at https://osf.io/smvzy/. For each experiment, analyses were conducted only after the entire sample was collected. Preregistration information is available for Experiments 2 (https://osf.io/pbdfc/), 4 (https://osf.io/5ketg/), and 7 (https://osf.io/w57ea/) and the Police-Involved Video Analysis (https://osf.io/bse9q/).

### Power Analysis, Recruitment, Participants, and Exclusions.

Prior to a power analysis, we conducted an initial experiment aiming for 25 people per cell or at least 100 people. (Experiment 2 was the first experiment conducted.) From this experiment, we calculated the required sample to detect a pairwise effect with power at 90% and α at 0.05. This analysis indicated we needed at least 98 participants per condition, which we rounded to 100. Given the possibility of incompletion by participants due to technical constraints (e.g., html5 video may not be supported on all browsers), we aimed for a sample that was 25% more than the recommended sample. Participants who, for any technical reason, were unable to see a video in their browser in a trial question were not passed on to the main experiment. We posted an advertisement for Experiments 1 to 7 on Amazon’s Mechanical Turk (M-Turk) and recruited US-based participants in exchange for $0.50. All data were collected from January 31, 2017 and July 11, 2018. Across Experiments 1 to 7, these data files represent 250 of 275, 105 of 115, 220 of 228, 348 of 390, 260 of 280, 308 of 330, and 425 of 505 completed experiments to participants recruited. In Experiment 8, conducted at a field laboratory, 482 people were approached, 217 qualified, and 203 completed the experiment.

### Police-Involved Video Analysis.

On June 15, 2018, we queried the YouTube API for the following search terms: body worn camera video release, body cam police, body cam footage, body cam arrest, body cam court, raw body cam, dash cam traffic stop, dash cam arrest, dash cam suspect, dash cam footage release, dash cam officer, dash cam department. Next, we collected videos from police video aggregation YouTube channels (e.g., PoliceActivity). This search yielded 750 videos. We removed duplicates, non–officer-involved videos, and videos that did not meet YouTube’s standards for copyright and graphic content.

We recruited Amazon’s M-Turk Masters (those with 99% approval rates on more than 100 human intelligence tasks, which are short, complex tasks that a computer is unable to perform). We paid $8 per hour. Originally, we assumed there would be high agreement in the coding for the videos, and we set a criterion that videos whose proportion scores were within 2% of each other would be averaged across each of our target variables (number of times on screen, length of time on screen); 56% of the videos met this criterion. Those videos with lower agreement tended to be of greater duration, suggesting the possible intrusion of coder fatigue in the case of longer videos. To correct for this problem, B.L.T. coded those videos for which there was a lack of agreement and substituted these results into the final analysis. To rule out the possibility that the author-coded videos introduced systematic bias due to knowledge of the hypotheses, we compared the author-coded videos to the M-Turk–coded videos and found no systematic variation in their codings of proportion of time spent on screen, *t* = 0.98, *P* = 0.32; time per appearance, *t* = 0.33, *P* = 0.74; or the number of body cam (vs. dash cam) videos coded, *t* = 0.98, *P* = 0.33.

### Stimuli.

Videos in Experiments 1, 4, 5, and 8 were collected from YouTube. Videos in Experiments 2 and 3 were filmed simultaneously by three Zoom QD HD Handycam digital video recorders. The videos in Experiments 6 and 7 were filmed with two Apple iPhone cameras. The cameras were worn at chest level in the body cam-obscured condition. In the body cam-visible condition, the cameras were attached to the left shoulder of the actors. In the dash cam condition, the videos were shot so that the actor’s entire body was present in the video. The videos were filmed separately and then edited to have the same start and stop points. Actors rehearsed their movements multiple times so that they used the same movement in each scene.

### Experiment 1.

Demographic information was not collected in this experiment. Participants indicated their judgments of intention (“The officer intentionally [broke the car window/shot the suspect]”; 1 = strongly disagree, 7 = strongly agree), blame (“How much blame does the officer deserve [for breaking the car window/shooting the suspect]”; 1 = none at all, 7 = a great deal), and recommended punishment (“How much should the officer be punished for [breaking the car window/shooting the suspect]”; 1 = not at all, 7 = a great deal).

### Experiments 2 and 3.

Demographic information was not collected in these experiments. In neither experiment was there significant variation as a function of which actor was wearing the body cam, *F*(1, 101) = 0.22, *P* = 0.64; *F*(1, 216) = 0.53, *P* = 0.47; nor any interaction between the actor wearing the body cam and the visual perspective, *F*(1, 101) = 0.003, *P* = 0.96; *F*(1, 216) = 0.51, *P* = 0.48. Thus, all results were collapsed and analyzed by the single factor of body cam versus dash cam.

### Experiment 4.

Participants (mean age = 37.16 y; SD = 12.26 y; 63% female; 83% white) either read the perspective-taking condition instruction: “In preparing for this task, take the perspective of the police officer in each video. Try to understand what they are thinking. What are their interests and purpose in the situation? Try to imagine what you would be thinking if you were in their shoes,” (14) or the control condition instruction, which only informed them they would make judgments about police-involved videos. Intentionality was measured with two questions (Movie S1: “The officer intentionally broke the car window”; “The officer intended to break the car window”; 1 = strongly disagree, 7 = strongly agree; *r* = 0.71; Movie S2: “The officer intentionally shot the suspect”; “The officer intended to shoot the suspect”; 1 = strongly disagree, 7 = strongly agree; *r* = 0.62). Details on manipulation check ratings and results appear in *SI Appendix*.

We controlled for participants’ concern about crime (“I worry often about being a victim of crime”), attitudes toward police officers (“I trust the police”; “The police are fair”; 1 = strongly disagree, 7 = strongly agree; *r* = 0.85), political orientation (1 = Liberal, 7 = Conservative; 1 = Democrat, 7 = Republican; *r* = 0.74), whether they identified as white and their age, sex, and income. These variables were submitted to a 2 (perspective taking) × 2 (body cam vs. dash cam) × 2 (incident) repeated-measures analysis of covariance, with the latter factor being within-subject, along with the seven covariates. We noted the main effect body cam versus dash cam, *F*(1, 340) = 31.27, *P* < 0.001, such that body cam (mean = 4.74, SD = 1.59) showed lower intentionality judgments than dash cam (mean = 5.38, SD = 1.53). The within-subject factor of incident was significant, *F*(1, 340) = 8.53; *P* = 0.004, such that intentionally judgments for the car window video (mean = 5.53; SD = 1.51) were higher than the judgments for the shooting (mean = 4.60; SD = 1.53). There were no interaction effects between the fixed factors (*P*s > 0.25). Of the covariates, only the concern about crime was significant in the model, *F*(1, 340) = 6.25, *P* = 0.004. We found no interactive effect of concern for crime on any combination of factors (*P*s > 0.80). For ease of interpretation, we combined the intentionality ratings across the two videos (α = 0.58).

### Experiment 5.

Participants (mean age = 34.92 y; SD = 10.54 y; 38% female; 84% white) in the neutral incident valence condition read the nonofficer was a suspect. The negative incident valence condition added information that “a baby was in the backseat, and was injured by the broken glass,” and “the person shot was innocent, and a father of two.”

A repeated-measures ANOVA revealed a main effect of the video, *F*(1, 256) = 69.98, *P* < 0.001, such that intentionally judgments for breaking the car window (mean = 6.03; SD = 1.41) were higher than for the shooting (mean = 5.22; SD = 1.33). There was no main effect of incident valence on the intentionality ratings. The interaction of the within-subject factor (video) with incident valence, visual perspective, and the interaction of these two manipulations was not significant (*P*s > 0.25). For ease of interpretation, we combined intentionality ratings across the videos, and presented a single measure of intentionality (α = 0.54).

### Experiments 6 and 7.

Participants in Experiments 6 (mean age = 34.88 y; SD = 10.69 y; 55% female; 80% white) and 7 (mean age = 35.50 y; SD = 11.57 y; 55% female; 78% white) were shown all scenarios in random order. For each scenario, they randomly saw one type of visual perspective: body cam-obscured, body cam-visible, body cam-face (Experiment 7 only), or dash cam.

### Experiment 8.

Participants were recruited from the University of Chicago field laboratories between October 22, 2017 and November 18, 2017. A total of 482 people were approached. Of those, 217 agreed to complete the experiment, and 203 completed the entire instrument and are included in the final data and analysis. Materials seen by participants (45% female; 79% white) are available in *SI Appendix*. Participant age was not collected, other than verification of being age 18 y or older.

## Supplementary Material

Supplementary File

Supplementary File

Supplementary File

Supplementary File

Supplementary File

Supplementary File

Supplementary File

Supplementary File

Supplementary File

Supplementary File

Supplementary File

Supplementary File

Supplementary File

Supplementary File

Supplementary File

Supplementary File

Supplementary File

Supplementary File

Supplementary File

Supplementary File

Supplementary File

Supplementary File

Supplementary File

Supplementary File

Supplementary File

Supplementary File

Supplementary File

Supplementary File

Supplementary File

Supplementary File

Supplementary File

Supplementary File

Supplementary File

Supplementary File

Supplementary File

Supplementary File

Supplementary File

Supplementary File

Supplementary File

Supplementary File
