# Supplementary material for: Body camera footage leads to lower judgments of intent than dash camera footage
Source: Proc Natl Acad Sci U S A. 2019 Jan 7;116(4):1201–6. doi: 10.1073/pnas.1805928116 (PMC6347687; doi:10.1073/pnas.1805928116)

PNAS
www.pnas.org

Supplementary Information for

Body camera footage leads to lower judgments of intent than dash camera footage

Broderick L. Turner, Eugene M. Caruso, Mike Dilich and Neal J. Roese

Broderick Turner
E-mail: broderick.turner@kellogg.northwestern.edu

This PDF file includes:

Figs. S1 to S4
Tables S1 to S4
Captions for Movies S1 to S30
Captions for Databases S1 to S9
References for SI reference citations

Other supplementary materials for this manuscript include the following:

Movies S1 to S30
Databases S1 to S9

www.pnas.org/cgi/doi/10.1073/pnas.1805928115

Supporting Information Appendix. This section includes all videos and data used for all experiments in "Body camera footage leads to lower judgments of intent than dash camera footage." This section also includes additional details omitted from the manuscript because of space constraints.

Additional data table S1 (DatasetExperiment1.csv)
Experiment 1 Data

Additional data table S2 (DatasetExperiment2.csv)
Experiment 2 Data

Additional data table S3 (DatasetExperiment3.csv)
Experiment 3 Data

Additional data table S4 (DatasetExperiment4.csv)
Experiment 4 Data

Additional data table S5 (DatasetExperiment5.csv)
Experiment 5 Data

Additional data table S6 (DatasetExperiment6.csv)
Experiment 6 Data

Additional data table S7 (DatasetExperiment7.csv)
Experiment 7 Data

Additional data table S8 (DatasetExperiment8.csv)
Experiment 8 Data

Additional data table S9 (DatasetPoliceVideoAnalysis.csv)
Police-Involved Video Analysis Data

Additional Analysis of Visual Salience

Refer to Figure S1. To visually represent the impact of visual salience on intentionally ratings, we plotted the proportion of on-screen time for the focal actor against mean intentionality rating ($\pm$CI) for each scenario presented in our experiments. Graph (A) summarizes mean intentionality ratings across all videos. Graphs for specific videos are as follows: (B) non-scripted footage of an officer breaking a car window used in Experiments 1, 4, and 5, (C) non-scripted footage of a police shooting used in Experiments 1, 4, and 5, (D) non-scripted footage of another police shooting used in Experiment 1, (E) scripted bump between two female actors used in Experiment 2, (F) scripted bump between two male actors used in Experiment 3, (G) scripted scene of actor dropping a magazine, (H) scripted scene of actor tipping over a cup of water, (I) scripted scene of actor pulling a stuffed animal off of a shelf, (J) scripted scene of actor kicking over a trashcan, and (K) an extended version of video (B) that includes the vehicle driving away and then crashing.

Experiment 1

Sample recruitment and exclusions. We posted an advertisement for this experiment to US residents on Amazon's Mechanical Turk in exchange for $0.50 for 24 hours between April 2, 2017, and April 3, 2017. We did not collect any demographic information. To account for the possibility of non-completion, we allowed for up to 300 participants. In total, 275 accepted the HIT. The consent form specified that participants could withdraw consent at any time by exiting an incomplete survey; therefore, only the participants who completed the survey in its entirety provided the ongoing consent necessary for us to analyze their data (in this and all subsequent experiments). Responses from those participants who completed the entire survey constitute the 250 observations provided in the data file (Experiment 1 Data). No other participants were removed, and no attention checks were used in this experiment.

Stimuli and procedures. The videos in this experiment were collected from YouTube. For each of the 3 incidents, videos included both the body cam and dash cam versions of the same incident. Two videos depicted a police-involved shooting. A third video was edited from a longer incident and showed the portion of a police video in which an officer broke a car window. Participants were randomly assigned to the body cam or dash cam conditions, and saw the same type of video for each scenario. Participants then answered items that measured their judgments of intentionality ("The officer intentionally [broke the car window/shot the suspect]"; 1= Strongly Disagree, 7 = Strongly Agree), blame ("How much blame does the officer deserve [for breaking the car window/shooting the suspect]"; 1=None at all, 7 = A great deal), and recommended punishment ("How much should the officer be punished for [breaking the car window/shooting the suspect]"; 1 = Not at all, 7 = A great deal). The means and standard deviations of each of these ratings are presented in the Table S3.

Experiment 2

Sample recruitment and exclusions. We posted an advertisement for this experiment to US residents on Amazon's Mechanical Turk in exchange for $0.50 and kept the experiment open for 24 hours between January 31, 2017, and February 1, 2017. We did not collect any demographic information. We left the survey open until we collected 115 responses. Responses from those participants who completed the entire survey constitute the 105 observations provided in the data file (Experiment 2 Data).

Stimuli. The videos for this experiment were filmed simultaneously by 3 Zoom QD HD Handy-cam digital video recorders. The cameras were worn at chest level by both actors. The actors were of similar height, build, and ethnicity. Both actors were male, and both had the same hair color. The dash cam was positioned 3 meters away from the incident and was 1.5 meters off the ground. The video depicted the two actors bumping into each other. Thus, there were three versions of the video: Two videos were captured by the body cam of each wearer, and one video captured the dash cam perspective. All videos were filmed indoors under florescent lighting.

Procedure. All participants read "In the next video you will see an interaction between two people." All participants then viewed videos of Person A and Person B and were asked to identify both to verify that their web browser showed the videos correctly. In the Body Cam condition, Person B was identified as "Person B body cam" and footage from their body cam was shown on a loop. In the dash cam condition, Person B was identified by a label on the video that read "Person B." To control for possible biased reaction toward either of the two actors, participants were randomly assigned to a second condition in which the first actor was Person A (and the second actor was Person B) or the first actor was Person B (and the second actor was Person A), as shown in Figure S2. After verification that the videos worked on their browsers, participants viewed either a body cam or dash cam video (Actor 1 or 2), and were asked to make judgments on the intentionality of Person B.

Experiment 3

Sample recruitment and exclusions. We posted an advertisement for this experiment to US residents on Amazon's Mechanical Turk in exchange for $0.50 and kept the experiment open for 24 hours between February 23, 2017, and February 24, 2017. We did not collect any demographic information. We collected 228 responses. Responses from those participants who completed the entire survey constituted the 220 observations provided in the data file (Experiment 3 Data).

Stimuli. The videos for this experiment were recorded with 3 Zoom QD HD Handy-cam digital video recorders. The cameras were worn at chest level by both actors. The actors were of similar height, build, and ethnicity. Both actors were female, and both had the same hair color. The dash camera was positioned 3 meters away from the incident and was 1.5 meters off the ground. The video depicted the two actors bumping into each other. Thus, there were three versions of the video: Two videos were captured by the body cam of each wearer, and one video captured the dash cam perspective. All videos were filmed outdoors under natural lighting.

Procedure. Experiment 3 followed the same procedure as Experiment 2.

Experiment 4

Sample recruitment and exclusions. We posted an advertisement for this experiment to US residents on Amazon's Mechanical Turk in exchange for $0.50 and kept the experiment open for 24 hours between July 10, 2018, and July 11, 2018. We left the survey open until we collected 402 responses. Complete responses from those participants constitute the 348 observations (Age = 37.16, SD = 12.26, 63% Female, 83% White) provided in the data file (Experiment 4 Data).

Stimuli and Procedures. Two videos from Experiment 1 (the window breaking video and the police shooting 2 video) were used as the stimuli for this experiment. In the control conditions, participants read, "You will see videos of police officers from either their body cam or dash cam. You will then make judgments about these videos." In the perspective-taking conditions, the participants read instructions adapted from past research on perspective taking (1): "In preparing for this task, take the perspective of the police officer in each video. Try to understand what they are thinking. What are their interests and purpose in the situation? Try to imagine what you would be thinking if you were in their shoes."

We measured intentionality after each video with two questions (Video 1: "The officer intentionally broke the car window"; "The officer intended to break the car window"; 1 = Strongly Disagree, 7 = Strongly Agree; $r = 0.71$; Video 2: "The officer intentionally shot the suspect"; "The officer intended to shoot the suspect"; 1 = Strongly Disagree, 7 = Strongly Agree; $r = 0.62$). Participants then answered manipulation check questions ("In the videos, I put myself in officer's shoes"; "In the videos, I took the perspective of the officer"; 1 = Strongly Disagree, 7 = Strongly Agree; $r = 0.75$). We also collected ratings of participants' perceived objectivity ("While watching the videos, I was objective in my judgments about the officer"; "I maintained objectivity while watching each video"; 1 = Strongly Disagree, 7 = Strongly Agree; $r = 0.79$) and their motivation for being accurate ("I wanted to be accurate in judging what happened in each video"; 1 = Strongly Disagree, 7 = Strongly Agree) and fair ("I wanted to be fair in judging what happened in each video"; 1 = Strongly Disagree, 7 = Strongly Agree).

Extended Results. To check our manipulation, we submitted each variable to a 2 (perspective-taking) X 2 (body cam vs. dash cam) between-participant ANOVA. The perspective-taking manipulation influenced participants' self-reported tendency toward taking the perspective of the officer, and also increased their perceived objectivity, but did not impact their perceived accuracy or fairness (Ps > 0.50). Specifically, those in the perspective-taking condition indicated that they took the officer's perspective (M = 5.81, SD = 1.13) to a greater extent than those in the control condition (M = 4.89, SD = 1.31), $t(346) = 7.03$, $P < 0.001$. Further, those in the perspective-taking condition reported being more objective in their judgments (M = 5.83, SD = 0.96) than those in the control condition (M = 5.25, SD = 1.09), $t(346) = 5.28$, $P < 0.001$.

Finally, we controlled for participants' concern about crime ("I worry often about being a victim of crime"; 1 = Strongly Disagree, 7 = Strongly Agree), attitudes toward police officers ("I trust the police"; "The police are fair"; 1 = Strongly Disagree, 7 = Strongly Agree; $r = 0.85$), political orientation (1= Liberal; 7 = Conservative; 1 = Democrat, 7 = Republican; $r = 0.74$), whether they self-identified as White (0 = No; 1 = Yes), age, gender, and income (1 = Less than $10K USD per year; 2 = 10 to 19.99; 3 = 20 to 29.99; 4 = 30 to 39.99; 5 = 40 to 49.99; 6 = 50 to 59.99; 7 = 60 to 69.99; 8 = 70 to 79.99; 9 = 80 to 89.99K; 10 = 90 to 99.99; 11 = 100 to 149.99; 12 = 150K or more).

These variables were submitted to a 2 (perspective taking) X 2 (body cam vs. dash cam) X 2 (incident) measures ANCOVA, with the latter factor being within-subject, along with the seven covariates. We noted the main effect body cam vs. dash cam, $F(1, 340) = 31.27$, $P < 0.001$, such that those in the body cam condition (M = 4.74, SD = 1.59) showed lower intentionality judgments than in the dash cam condition (M = 5.38, SD = 1.53). Also, the within-subject factor of incident was significant, $F(1, 340) = 8.53$, $P = 0.004$, such that intentionally judgments for the car window video (M = 5.53, SD = 1.51) were higher than the judgments for the shooting (M = 4.60, SD = 1.53). There was no interaction between perspective-taking and incident, $F(1, 340) = 0.10$, $P = 0.75$, nor between visual perspective and incident, $F(1, 340) = 0.35$, $P = 0.25$, nor between perspective-taking and visual perspective, $F(1, 340) = 1.29$, $P = 0.25$. Of the covariates, only the concern about crime variable was significant in the model, $F(1, 340) = 6.25$, $P = 0.004$. For ease of interpretation, we combined the intentionality ratings across the 2 videos $\alpha = 0.58$) and present the full model with this dependent variable in Table S4. Finally, to test the possible interactive effects of concern for crime on the model factors, we ran the following linear model predicting intent, Intent= $\beta_0 + (\beta_1 P \text{ X } \beta_2 V \text{ X } \beta_3 I \text{ X } \beta_4 C) + \epsilon$; where P = Perspective Taking Manipulation (0 = Control, 1 = Perspective Taking Manipulation), V = Video Type (0 = Body Cam, 1 = Dash cam), I = Incident (0 = Window Breaking, 1 = Police Shooting), and C = Concern About Crime (continuous). In this model, concern about crime held no interactive effect on any combination of factors (Ps $> .80$), while the main effect of body cam vs. dash cam remained significant, $\beta = -0.57$, SE = 0.23, $t = 37.62$, $P < 0.001$.

Experiment 5

Sample recruitment and exclusions. We posted an advertisement to US residents on Amazon's Mechanical Turk in exchange for $0.50 and kept the experiment open for 24 hours between May 16, 2017 and May 17, 2017. We collected 280 responses (Age = 34.92, SD = 10.54, 38% Female, 84% White). Responses from those participants who completed the entire survey constituted the 260 observations provided in the data file (Experiment 4 Data).

Stimuli and Procedures. The same videos from Experiment 4 were used as the stimuli for this experiment. The valence of the incident was manipulated with verbal descriptions to be either neutral or negative. In the neutral condition, the person who was not the police officer was identified as a suspect. The negative outcome condition informed participants that "a baby was in the backseat, and was injured by the broken glass," and "the person shot was innocent, and a father of two." After each intention judgment, participants answered a manipulation check question ("The outcome of this video was"; 1 = Negative, 7 = Positive).

Extended Results. To check the manipulation of incident valence, we submitted the manipulation check measure to a 2 (incident valence) X 2 (body cam vs. dash cam) between-participant ANOVA. We found that incident valence had an effect, such that the negative outcome was rated as more negative (M = 3.29. SD = 1.63) than the positive outcome (M = 3.84, SD = 1.53) $F(1, 256) = 7.98$, $P = 0.005$. There was no main effect of body cam (vs. dash cam), nor was the interaction significant (Ps > 0.44), thus suggesting that the manipulation was successful.

Next, we ran a repeated measures ANOVA that found that there was a main effect of intentionality judgments on the video that was presented, $F(1, 256) = 69.98$, $P < 0.001$, such that the average intentionally judgment for breaking the car window (M = 6.03, SD = 1.41) was higher than the judgment for the shooting (M = 5.22, SD = 1.33). There was no main effect of the incident valence manipulation on the intentionality ratings. The interactions between outcome valence, $F(1, 256) = 0.001$, $P = 0.98$, visual perspective as body cam or dash cam, $F(1, 256) = 1.33$, $P = 0.25$, and the interaction of these two manipulations, $F(1, 256) = 0.07$, $P = 0.78$, were not statistically significant. For ease of interpretation, we combined intentionality ratings across the videos, and presented a single measure of intentionality ($\alpha = 0.54$).

Experiment 6

Sample recruitment and exclusions. We posted an advertisement to US residents on Amazon's Mechanical Turk in exchange for $0.50 and kept the experiment open for 24 hours between April 29, 2017, and April 30, 2017. We collected 330 responses. Responses from those participants (Age = 34.88, SD = 10.69, 55% Female, 80% White) who completed the entire survey constituted the 308 observations provided in the data file (Experiment 6 Data).

Stimuli and procedures. The videos for this experiment were filmed with two iPhone cameras. The cameras were worn at chest level in the body cam-obscured condition. In the body cam-visible condition, the cameras were attached to the left shoulder of the actors. In the dash cam condition, the videos were shot so that the actor's entire body was present in the video. The videos were filmed separately after having each actor rehearse the movement multiple times so that they used the same movement in each scene. The scenes showed 1) a man tipping over a cup of water, 2) a woman kicking over a trashcan, 3) a man pulling a Hello Kitty stuffed animal off of a shelf, and 4) a woman dropping a magazine. All videos were filmed indoors under fluorescent lighting.

Participants were shown all scenarios in a random order. For each scenario, they were randomly presented with one type of video: body cam-visible, body cam-obscured, or dash cam. The results were combined across scenarios and analyzed per video type condition as described in the main body of the paper.

Experiment 7

Sample recruitment and exclusions. We posted an advertisement to US residents on Amazon's Mechanical Turk in exchange for $0.50 and kept the experiment open for 24 hours between July 11, 2018, and July 12, 2018. We collected 478 responses. Responses from those participants (Age = 35.50, SD = 11.57, 55% Female, 78% White) who completed the entire survey constituted the 425 observations provided in the data file (Experiment 7 Data).

Stimuli and procedures. We used the same videos from Experiment 6. However, to create the new body cam-face condition, we edited the body cam-obscured video to include a picture of the actor's face along with their name, both of which were visible for the duration of the video (see Figure S3 for an example). Participants were shown videos in a random order. The results were combined across scenarios and analyzed per video type condition as described in the main body of the paper.

Experiment 8

Sample recruitment and exclusions. Participants were recruited via a field research site operated by the Center for Decision Research at The University of Chicago between October 22, 2017 and November 18, 2017. Four hundred eighty-two people were approached. Of those, 217 indicated they were over 18 and qualified for jury duty in Illinois, and agreed to complete the experiment. Of those 203 (45% Female, 79% White) completed the entire instrument and are included in the final data and analysis (Experiment 8 Data).

These participants were randomly assigned to one of three conditions. In all conditions, they saw the actual redacted police accident report (Figure S4). In the report only condition, this is all that they saw. In the body cam-report condition, they also saw the body cam video of the event described in the police report; in the dash cam-report condition, they also saw the video cam version of the incident. In this incident, which shows an extended version of the window breaking incident used in Experiments 1, 4, and 5, an officer approaches a non-moving vehicle. He alerts the driver and breaks the driver's window with a Billy club. The driver then wakes up, drives off, and crashes into a pole.

Stimuli and procedures. Participants judged how intentional the officer's actions were. They were then shown a list of possible crimes that an officer could be charged with under the Illinois penal code. This list of possible crimes was developed in consultation with a criminal attorney with experience in litigation of police officers. Participants were given the following instructions for indictment decisions:

> All crimes are taken from the codified Illinois Compiled Statutes (ILCS).
>
> After viewing the evidence, you must now decide which, if any of the following crimes will you choose to charge the officer in question:
>
> ***Assault.*** (720 ILCS 5/12-1) (from Ch. 38, par. 12-1) A person commits an assault when, without lawful authority, he or she knowingly engages in conduct which places another in reasonable apprehension of receiving a battery.
>
> ***Battery.*** (720 ILCS 5/12-3) (from Ch. 38, par. 12-3) A person commits battery if he or she knowingly without legal justification by any means (1) causes bodily harm to an individual or (2) makes physical contact of an insulting or provoking nature with an individual.
>
> ***Aggravated Battery.*** (720 ILCS 5/12-3.05) (was 720 ILCS 5/12-4). (a) Offense based on injury. A person commits aggravated battery when, in committing a battery, other than by the discharge of a firearm, he or she knowingly does any of the following: (1) Causes great bodily harm or permanent disability or disfigurement.
>
> ***Official Misconduct.*** (720 ILCS 5/33-3) (from Ch. 38, par. 33-3) (a) A public officer or employee or special government agent commits misconduct when, in his official capacity or capacity as a special government agent, he or she commits any of the following acts: (1) Intentionally or recklessly fails to perform any mandatory duty as required by law; or (2) Knowingly performs an act which he knows he is forbidden by law to perform; or (3) With intent to obtain a personal advantage for himself or another, he performs an act in excess of his lawful authority; or

(4) Solicits or knowingly accepts for the performance of any act a fee or reward which he knows is not authorized by law

For each charge, participants made binary judgment of indict or do not indict.

References

1. Galinsky AD, Maddux WW, Gilin D, White JB (2008) Why it pays to get inside the head of your opponent: The differential effects of perspective taking and empathy in negotiations. *Psychological Science* 19(4):378–384.

Table S1. Description of Stimulus Videos

File. Incident	Type	VP	Experiment	Length	Prop	Appear	Time Per
**Movie S1. Breaking car window**	Police	BC	1,4,5	43	0.24	13	3
**Movie S2. Breaking car window**	Police	DC	1,4,5	43	0.95	1	3
**Movie S3. Police shooting 1**	Police	BC	1	10	0.95	2	3
**Movie S4. Police shooting 1**	Police	DC	1	10	1	1	3
**Movie S5. Police shooting 2**	Police	BC	1,4,5	10	0.4	3	2
**Movie S6. Police shooting 2**	Police	DC	1,4,5	10	1	1	2
**Movie S7. Bump-female**	Scripted	BC1	2	6	0	0	0.81
**Movie S8. Bump-female**	Scripted	BC2	2	6	0.17	1	1.33
**Movie S9. Bump-female**	Scripted	DC	2	6	1	1	1.33
**Movie S10. Bump-male**	Scripted	BC1	3	6	0.04	1	69
**Movie S11. Bump-male**	Scripted	BC2	3	6	0.04	1	10
**Movie S12. Bump-male**	Scripted	DC	3	6	1	1	10
**Movie S13. Dropping magazine**	Scripted	BCO	6,7	3	0	0	0
**Movie S14. Dropping magazine**	Scripted	BCF	7	3	0	0	0
**Movie S15. Dropping magazine**	Scripted	BCV	6,7	3	1	1	0
**Movie S16. Dropping magazine**	Scripted	DC	6,7	3	1	1	0
**Movie S17. Tipping over cup**	Scripted	BCO	6,7	2	0	0	0.25
**Movie S18. Tipping over cup**	Scripted	BCF	7	2	0	0	0.25
**Movie S19. Tipping over cup**	Scripted	BCV	6,7	2	1	1	0
**Movie S20. Tipping over cup**	Scripted	DC	6,7	2	1	1	0

Note. File.Incident = supplementary information file name and description of filmed incident, Type = type of video (Police = real police footage, Scripted = scripted experimental video), VP = visual perspective (BC = body cam, BC1 = body cam-actor 1, BC2 = body cam-actor 2, BCO = body cam-obscured, BCV = body cam-visible, BCF = body cam-face, DC = dash cam), Experiment = experiment number, Length = total length of each video in seconds, Prop = proportion of time the focal actor is visible in each video, Appear = number of appearances of the focal actor, Time Per = time per appearance of the focal actor in seconds.

Table S2. Description of Stimulus Videos Continued

File. Incident	Type	VP	Experiment	Length	Prop	Appear	Time Per
**Movie S21. Pulling down stuffed animal**	Scripted	BCO	6,7	3	0	0	41
**Movie S22. Pulling down stuffed animal**	Scripted	BCF	7	3	0	0	41
**Movie S23. Pulling down stuffed animal**	Scripted	BCV	6,7	3	0.67	1	4.75
**Movie S24. Pulling down stuffed Animal**	Scripted	DC	6,7	3	1	1	2
**Movie S25. Kicking over trashcan**	Scripted	BCO	6,7	3	0	0	3
**Movie S26. Kicking over trashcan**	Scripted	BCF	7	3	0	0	3
**Movie S27. Kicking over trashcan**	Scripted	BCV	6,7	3	1	1	6
**Movie S28. Kicking over trashcan**	Scripted	DC	6,7	3	1	1	6
**Movie S29. Breaking car window-extended**	Police	BC	8	70	0.46	24	0.25
**Movie S30. Breaking car window-extended**	Police	DC	8	70	0.99	1	1

Note. File.Incident = supplementary information file name and description of filmed incident, Type = type of video (Police = real police footage, Scripted = scripted experimental video), VP = visual perspective (BC = body cam, BC1 = body cam-actor 1, BC2 = body cam-actor 2, BCO = body cam-obscured, BCV = body cam-visible, BCF = body cam-face, DC = dash cam), Experiment = experiment number, Length = total length of each video in seconds, Prop = proportion of time the focal actor is visible in each video, Appear = number of appearances of the focal actor, Time Per = time per appearance of the focal actor in seconds.

Fig. S1. Intentionality Judgment as a Function of Visual Salience

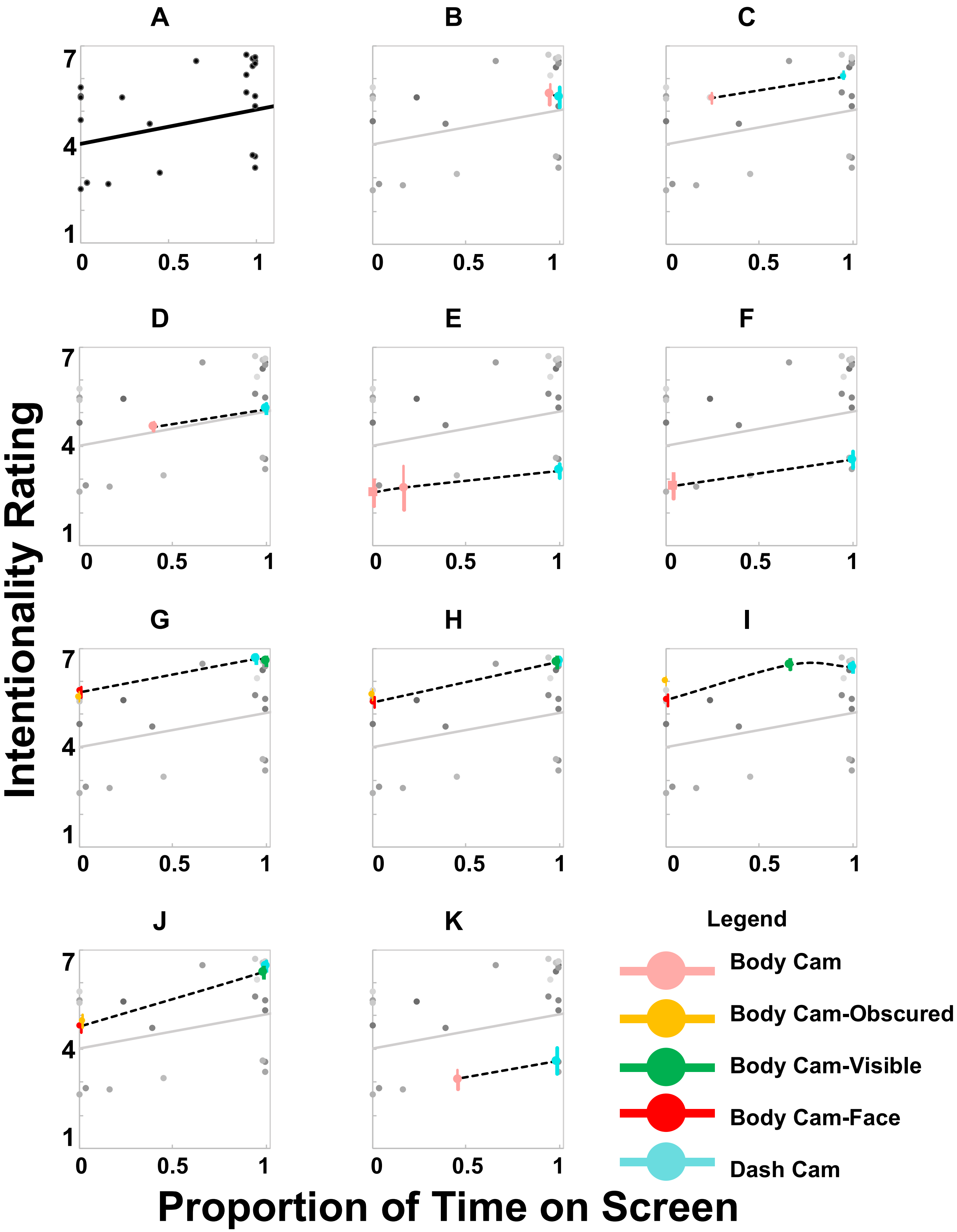

Table S3. Means (SDs) of Each Measure per Scene in Experiment 1

Item	Condition	Incident<br>Broken Window	Police Shooting 1	Police Shooting 2
Intention	Body cam	5.30 (1.76)***	5.54 (1.67)n.s.	4.52 (1.39)***
	Dash cam	6.18 (1.19)	5.44 (1.73)	5.24 (1.42)
Deserved	Body cam	2.43 (1.78)***	2.37 (1.64)**	3.21 (1.62)***
Blame	Dash cam	4.05 (2.15)	3.07 (1.99)	4.23 (1.84)
Recommended	Body cam	3.14 (2.17)***	2.99 (2.00)*	3.64 (1.68)***
Punishment	Dash cam	4.64 (2.15)	3.47 (1.96)	4.64 (1.80)

Df = 248, ****P* **< 0.05, **** ***P*** **< 0.01, ******P*** **< 0. 001, $^{\text{n.s.}}$ Not Significant**

Fig. S2. Example of Actor Identification in Experiments 2 and 3

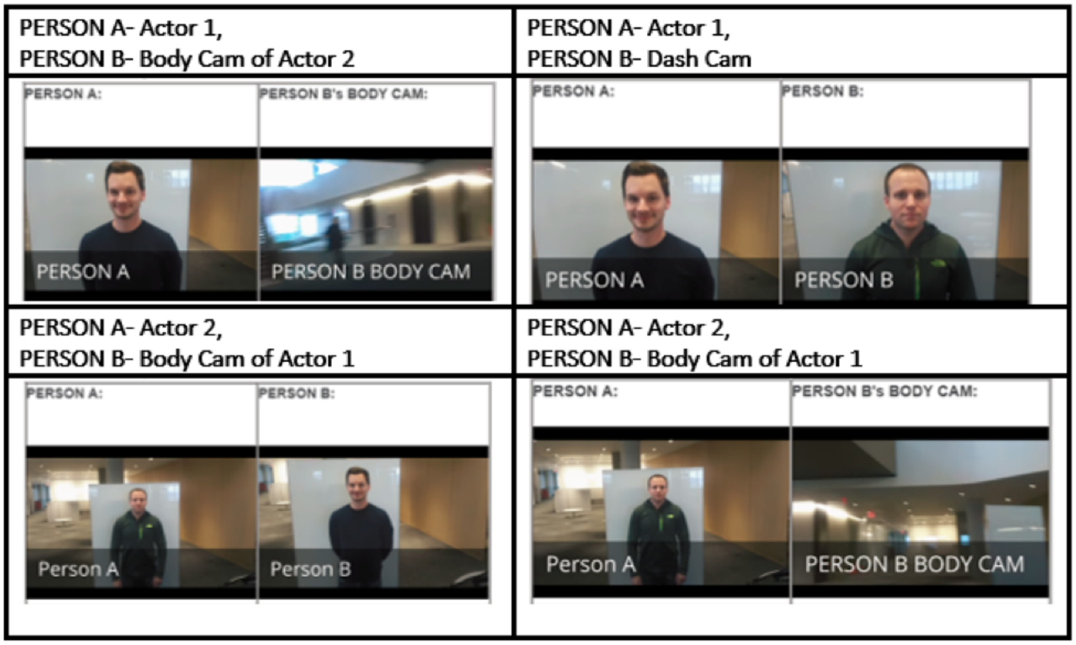

Table S4. Repeated Measures ANCOVA (Experiment 4)

	Mean (SD)	Mean Square	*F*	*P*
Perspective Taking (A)		0.83	0.38	0.54
Dash cam vs. Body cam (B)		68.07	31.27	< 0.001***
Incident (C)		18.56	8.53	0.004**
A*B		0.02	0.01	0.92
A*C		0.67	0.3	0.58
B*C		0.76	0.35	0.55
A*B*C		2.83	1.3	0.25
Crime Concern	4.31 (1.73)	13.6	6.25	0.01*
Police Attitude	4.85 (1.46)	6.27	2.88	0.09
Political Orientation	4.08 (1.73)	6.82	3.13	0.08
White (0= No, 1 = Yes)	0.83	7.57	3.48	0.06
Age	37.16 (12.25)	4.97	2.28	0.13
Gender (0= Male,1 = Female)	0.63	0.06	0.09	0.76
Income	6.29 (3.10)	1.71	0.79	0.37

Df = 346, ****P* **< 0.05, *****P* **< 0.01, *** ***P*** **< 0.001, $^{n.s.}$Not Significant**

Fig. S3. Example of Body Cam-Face Condition used in Experiment 7

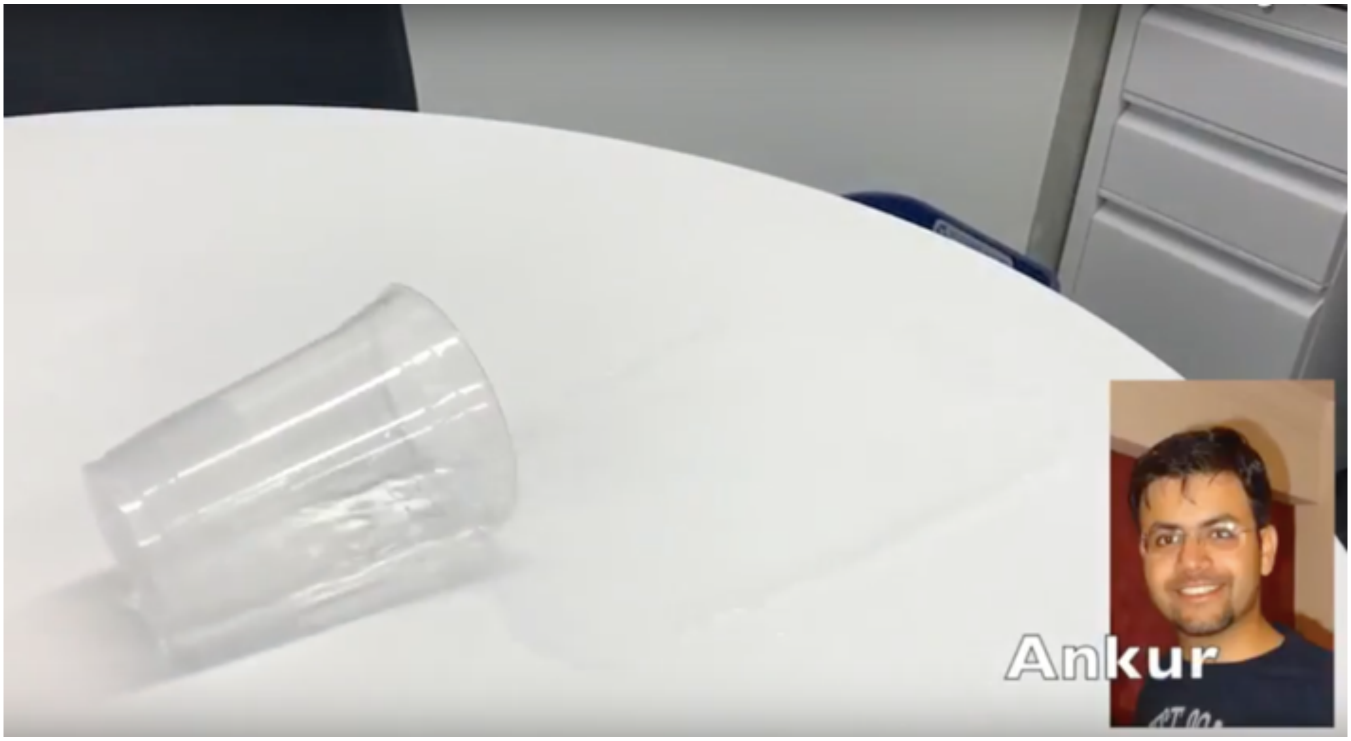

Fig. S4. Redacted Police Report used in Experiment 8

Law Enforcement an[redacted]
Form CR-3 (Rev. 1/1/2015)

Case ID [redacted]

[redacted] Crash ID

[redacted]

Driver of unit 1 was passed out behind the wheel of the vehicle in the 1800 block of University, facing South.
Driver was awoken by police.
Driver rapidly accelerated South through the intersection, failed to maintain a single lane and failed to control speed, and left the roadway.
Unit 1 drove through the grass on the West side of 1900 University, crossed an alley, went airborne, and crashed into a power pole.
The collision caused a live power line to break loose and fall on top of the vehicle. The power line arched to the vehicle and caused the vehicle to catch fire.
Driver then evaded police on foot, was caught, and placed under arrest for DWI.

IDENTIFICATION & LOCATION

*Crash Date (MM/DD/YYYY) 07/15/[redacted] — *Crash Time (24HRMM) 0759 — Case ID [redacted] — Local Us[redacted]

*County Name [redacted] — *City Name [redacted] — ☐ Outside City Limit

In your opinion, did this crash result in at least $1,000 damage to any one person's property? ☒ Yes ☐ No — Latitude (decimal degrees) — Longitude (decimal degrees) – [redacted]

ROAD ON WHICH CRASH OCCURRED

*1 Rdwy. Sys. LR — *Hwy. Num. — 2 Rdwy. Part 1 — Block Num. 1900 — 3 Street Prefix — * Street Name UNIVERSITY — 4 Street Suffix AVE

☐ Crash Occurred on a Private Drive or Road/Private Property/Parking Lot — ☐ Toll Road/Toll Lane [redacted] Speed Limit 40 — Const. Zone ☐ Yes ☒ No — Workers Present ☐ Yes ☒ No — Street Desc.

INTERSECTING ROAD, OR IF CRASH NOT AT INTERSECTION, NEAREST INTERSECTING ROAD OR REFERENCE MARKER

At Int. ☐ Yes ☒ No — 1 Rdwy. Sys. LR — Hwy. Num. — 2. Rdwy. Part 1 — Block Num. 2400 — 3 Street Prefix — Street Name 20TH — 4 Street Suffix ST

Distance from Int. or Ref. Marker 100 — ☒ FT ☐ MI — 3 Dir. from Int. or Ref. Marker N — Reference Marker — Street Desc. — RRX Num.

VEHICLE, DRIVER, & PERSONS

Unit Num. 1 — 5 Unit Desc. 1 — ☐ Parked Vehicle — ☐ Hit and Run — LP State [redacted] — LP Num. [redacted] — VIN 1[redacted]

Veh. Year 2002 — 6. Veh. Color SIL — Veh. Make PONTIAC — Veh. Model GRAND AM — 7 Body Style P4 — ☐ Pol., Fire, EMS on Emergency (Explain in Narrative if checked)

8 DL/ID Type 1 — DL/ID State [redacted] — DL/ID Num. [redacted] — 9 DL Class C — 10 CDL End. 96 — 11 DL Rest. 96 — DOB (MM/DD/YYYY) [redacted]

Address (Street, City, State, ZIP) 5418 79TH [redacted]

Person Num.	12 Prsn. Type	13 Seat Position	Name: Last, First, Middle<br>Enter Driver or Primary Person for this Unit on first line	14 Injury Severity	Age	15 Ethnicity	16 Sex	17 Eject	18 Restr.	19 Airbag	20 Helmet	21 Sol.	22 Alc. Spec.	Alc. Result	23 Drug Spec.	24 Drug Result	25 Drug Category
1	1	1	[redacted] Q	B	18	B	1	1	99	99	97	N	2		2	99	99

Not Applicable - Alcohol and Drug Results are only reported for Driver/Primary Person for each Unit.

☒ Owner ☐ Lessee — Owner/L[redacted] Name & [redacted]

ADDITIONAL DIAGRAM

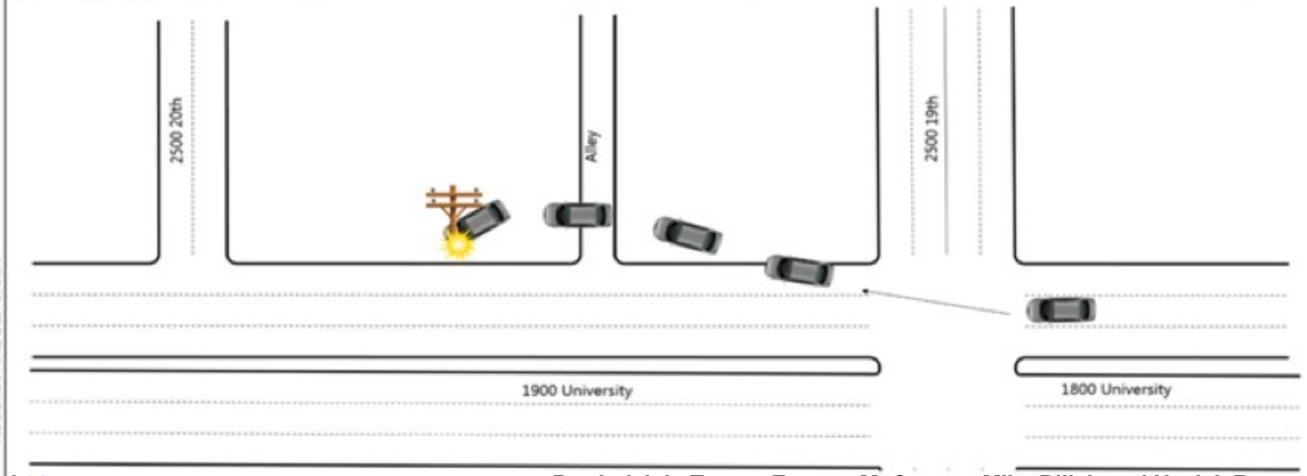

Supplement: Supplementary File [file pnas.1805928116.sapp.pdf]
